# Artificial intelligence-enabled multipurpose smart detection in active-matrix electrowetting-on-dielectric digital microfluidics

**DOI:** 10.1038/s41378-024-00765-7

**Published:** 2024-09-27

**Authors:** Zhiqiang Jia, Chunyu Chang, Siyi Hu, Jiahao Li, Mingfeng Ge, Wenfei Dong, Hanbin Ma

**Affiliations:** 1 College of Mechanical and Electrical Engineering, Changchun University of Science and Technology, Changchun, Jilin Province 130022 PR China; 2 CAS Key Laboratory of Bio-Medical Diagnostics, Suzhou Institute of Biomedical Engineering and Technology, Chinese Academy of Sciences, Suzhou, Jiangsu Province 215163 PR China; 3 Guangdong ACXEL Micro & Nano Tech Co. Ltd, Foshan, Guangdong Province 528000 PR China; 4 ACX Instruments Ltd, Cambridge, CB4 0WS UK

**Keywords:** Engineering, Nanoscience and technology

## Abstract

An active-matrix electrowetting-on-dielectric (AM-EWOD) system integrates hundreds of thousands of active electrodes for sample droplet manipulation, which can enable simultaneous, automatic, and parallel on-chip biochemical reactions. A smart detection system is essential for ensuring a fully automatic workflow and online programming for the subsequent experimental steps. In this work, we demonstrated an artificial intelligence (AI)-enabled multipurpose smart detection method in an AM-EWOD system for different tasks. We employed the U-Net model to quantitatively evaluate the uniformity of the applied droplet-splitting methods. We used the YOLOv8 model to monitor the droplet-splitting process online. A 97.76% splitting success rate was observed with 18 different AM-EWOD chips. A 99.982% model precision rate and a 99.980% model recall rate were manually verified. We employed an improved YOLOv8 model to detect single-cell samples in nanolitre droplets. Compared with manual verification, the model achieved 99.260% and 99.193% precision and recall rates, respectively. In addition, single-cell droplet sorting and routing experiments were demonstrated. With an AI-based smart detection system, AM-EWOD has shown great potential for use as a ubiquitous platform for implementing true lab-on-a-chip applications.

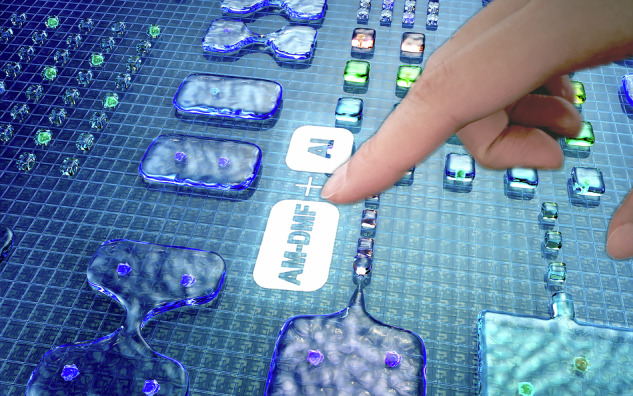

## Introduction

**Fig. 1:**
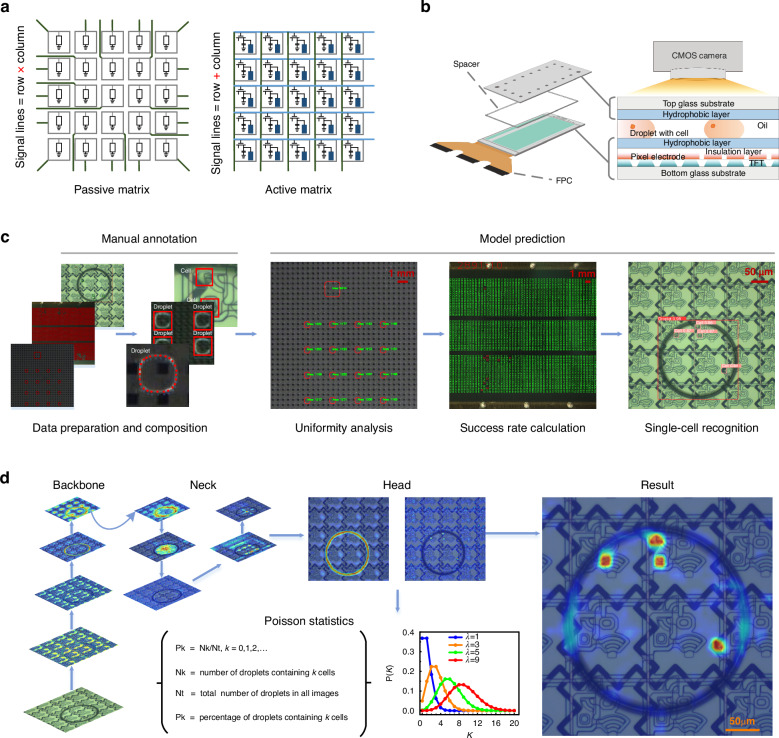
Artificial intelligence-enabled multipurpose smart detection process on an AM-EWOD chip. **a** Comparison between the driving schemes of PM and AM devices. **b** Structure of an AM-EWOD chip. **c** DL-enabled uniformity analysis, success rate calculation, and single-cell recognition. **d** DL workflow for droplet and single-cell detection

To generate and manipulate submicron-litre biosamples, powerful tools that are easy to operate, accurate, and multifunctional are needed^1,2^. To date, different technology platforms have been developed, including flow cytometry^3,4^, microwell microfluidics^5,6^, microdroplet microfluidics^7^, optical tweezers^8^, and digital microfluidics (DMF)^9–11^. The advantages of DMF systems over other platforms are that they can realize sample separation, real-time manipulation, and parallel in situ analyses, all while enabling the simultaneous manipulation of biosamples on a two-dimensional surface^12^. The high-throughput sample generation process demands a large number of electrodes driving droplets on a DMF chip. However, passive-matrix (PM) EWOD systems typically accommodate fewer than 200 electrodes, as each PM electrode is physically connected to a peripheral connector (Fig. 1a)^9^. The large number of associated connection lines limits the scalability of electrodes, posing a challenge in DMF research and development work. To address this issue, researchers have adopted active-matrix (AM) addressing, in which each pixel contains active transistors that act as switches and can be independently addressed by row and column driver lines (Fig. 1b)^13–15^. Several studies have been conducted using AM-DMF technology for molecular diagnosis^16^, proteomics analysis^17^, high-resolution concentration gradient preparation^18^, and parallel single-cell manipulation^1^ tasks.

AM-EWOD technology can generate and analyze thousands of discrete droplets surrounded by filler oil in parallel. However, determining and screening samples pose significant challenges for researchers, where manually selecting samples is an inefficient and inconsistent strategy. Traditional image processing techniques are mostly tailored for specific scenarios. They greatly rely on predefined distributions or manually designed features, which results in limited generalizability^19^. In recent decades, deep learning (DL) has made significant technological progress and has shown great potential for use as a powerful tool in an AM-EWOD system for multipurpose smart detection^20–24^. The applications of DL in droplet-based microfluidics are becoming increasingly widespread^25–31^. However, a limited number of studies have reported applications of DL technology in the AM-DMF field. Therefore, in this work, we addressed the automated biosample selection and determination problems on an AM-EWOD platform by using a range of DL models for different tasks (Fig. 1c).

The phenomenon of “necking” occurs during droplet splitting, and the length of the droplet neck affects the homogeneity of the volumes of the split droplets^32^. To achieve uniformity during droplet splitting, it is essential to ensure uniform control of the electrodes on both sides. However, it is challenging for an AM-DMF system to achieve highly uniform control of large-scale pixel electrodes because of the utilization of thin-film transistors (TFTs) as driving switches^33^. Therefore, we employed an image recognition method to assess droplet uniformity, allowing us to select droplets according to our needs under appropriate conditions, thereby compensating for the limitations imposed by the inherent challenges of the utilized system. High-throughput droplet generation provides extensive parallel data for biological analyses^34^. However, a trade-off exists between the success rate of droplet splitting and the number of droplets that can be generated and controlled per unit area in parallel^15,32^. Therefore, we propose a DL-based high-throughput droplet recognition method to iteratively design droplet-splitting paths and swiftly find an optimal solution. Furthermore, the method presented in this paper that uses DL-based high-throughput droplet recognition contributes to several advancements: optimizing chip packaging conditions, such as gap control and the thickness of the hydrophobic layer; conducting a statistical analysis of the single-cell sample generation rate, which is calculated as the ratio of the number of single-cell samples to the number of single droplets; and recording the positions of single droplets, thereby expediting the process of identifying single-cell samples. The generation of single-cell samples is essential for genomic^35,36^, transcriptomic^37,38^, proteomic^39,40^, and metabolomic^41,42^ studies. The technology that encapsulates cells in microfluidic droplets has been widely adopted^43–46^. However, separating single-cell samples from droplets becomes challenging after high-throughput droplets are generated with an AM-EWOD system. To address this issue, we introduce an improved YOLOv8 model for automatically recognizing single-cell samples (Fig. 1d), thus replacing manual sorting.

Our work can be summarized by the following research highlights.

**1. Uniformity analysis**: We used the U-Net model to evaluate the uniformity of the droplet volumes of the three tested strategies, replacing the manual evaluation process^47^. Our results revealed that droplets generated via the “one-to-two” method exhibited optimal uniformity.

**2. Success rate calculation**: We used the YOLOv8 model to calculate the success rate of the high-throughput droplet array generation procedure, thus replacing manual counting and addressing the inefficiency and inconsistency associated with this method.

**3. Single-cell recognition**: We used an improved YOLOv8 model to recognize single-cell samples, replacing manual sorting and automatically generating high-throughput single-cell samples.

## Results and discussion

### Uniformity analysis

**Fig. 2:**
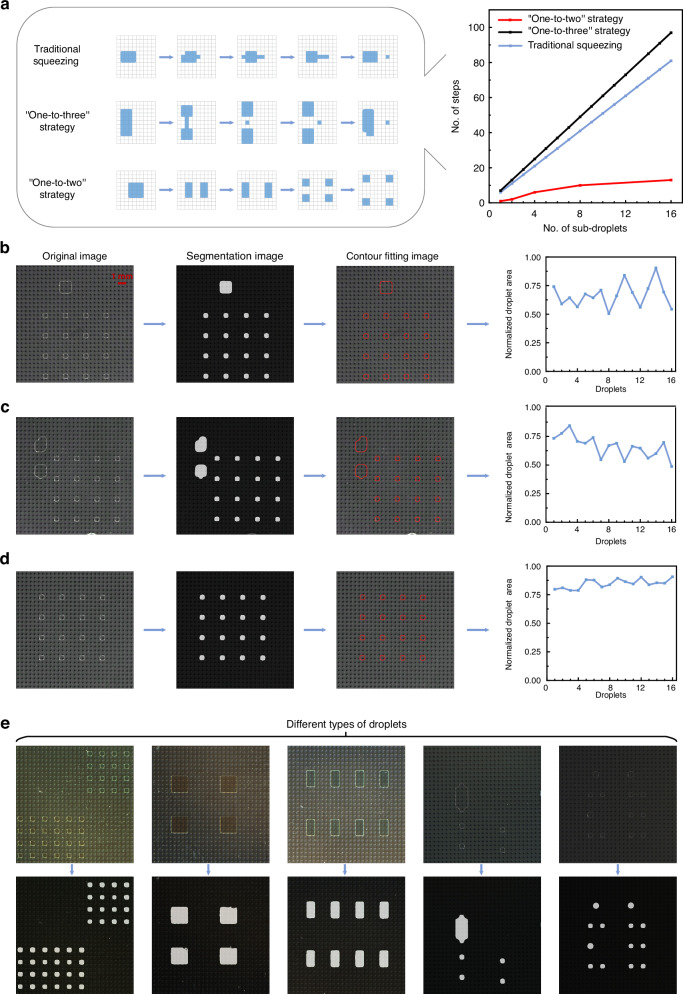
Performance analysis of different droplet generation strategies. **a** Schematic diagrams of the droplet generation processes implemented via the traditional squeezing, “one-to-three” strategy, and “one-to-two” strategy, as well as a graph showing the number of steps versus the number of subdroplets. **b** The original image, segmentation image, and contour-fitting image produced when performing droplet generation with the traditional squeezing and an area of 16 droplets. **c** The original image, segmentation image, and contour-fitting image produced when performing droplet generation with the “one-to-three” strategy and an area of 16 droplets. **d**. The original image, segmentation image, and contour-fitting image produced when performing droplet generation with the “one-to-two” strategy and an area of 16 droplets. **e** Model generalizability testing results obtained for different types of droplets with different sizes, colors, morphologies, and brightness levels

We evaluated the overall performance of three droplet generation strategies on an AM-EWOD chip, namely, the traditional squeezing strategy, the “one-to-three” strategy, and the “one-to-two” strategy. Schematic diagrams of the droplet-splitting paths of each strategy are presented in Fig. 2a on the left and detailed in Movies S1–S3. The targeted droplets, each of which possessed a volume of approximately 25 nanolitres, were controlled by an electrode. The microscope system is shown in Fig. S1. The traditional squeezing strategy is a classic droplet generation method for DMF systems. The “one-to-three” strategy involves a novel idea of splitting small droplets within a higher device aspect ratio. The “one-to-two” strategy efficiently generates many droplets within a short period. The right side of Fig. 2a illustrates the relationship between the number of generated subdroplets and the number of required steps. The traditional squeezing strategy required 4 steps to generate a droplet, with an additional wait time of 1 step to avoid merging with the previous droplet. To generate 16 subdroplets, each with a size of a 1 × 1 electrode and spaced by four electrodes, a total of 81 steps were needed. As each tearing step lasted 1 s, the entire process took 81 s. The “one-to-three” strategy required 2 steps to generate a droplet, with an additional wait time of 4 steps to avoid merging with the previous droplet. Under the same conditions, generating 16 droplets required 97 steps in total, resulting in a process duration of 97 s, with each step lasting 1 s. The “one-to-two” strategy generated droplets exponentially with the number of steps. To generate 16 droplets with sizes of 1 × 1 electrodes spaced by four electrodes, only 13 steps were needed. Each tearing step took 1 s, leading to a total processing time of 13 s. The fundamental relationships between the number of steps and the number of generated subdroplets are as follows:

1 $$\begin{array}{cc}{{\rm{Y}}}_{1}=\left(4+1\right){\rm{x}}+1 & 0 < {\rm{x}} < =16\end{array}$$

2 $$\begin{array}{cc}{{\rm{Y}}}_{2}=\left(2+4\right){\rm{x}}+1 & 0 < {\rm{x}} < =16\end{array}$$

3 $$\begin{array}{cc}{{\rm{Y}}}_{3}=1,2,6,10,13 & {\rm{x}}=1,2,4,8,16\end{array}$$

where *Y*_1_, *Y*_2_, and *Y*_3_ are the number of steps required for conducting droplet generation with the traditional squeezing strategy, “one-to-three” strategy, and “one-to-two” strategy, respectively. *x* is the number of subdroplets.

We used the U-Net segmentation algorithm to evaluate the uniformity of the three droplet generation strategies. We thoroughly discuss and compare the effectiveness of employing DL methods instead of traditional image processing techniques for the droplet segmentation task in the “Methods and materials” section. The workflow for conducting droplet segmentation with the U-Net segmentation algorithm is shown in Fig. S2. The experimental parameter settings are shown in Table S1. The loss functions used for the droplet segmentation model on the training and validation sets are shown in Fig. S3a, b, respectively. The mean intersection-over-union (mIoU) function used for the validation set is shown in Fig. S3c. Figure 2b shows the uniformity analysis process conducted for the 16 droplets generated by the traditional squeezing strategy, yielding a volume coefficient of variation of 2.61%. We used a CMOS camera to capture the original images. We subsequently obtained a mask image via the U-Net segmentation algorithm and obtained a contour-fitting image after applying further processing steps. The contour-fitting image demonstrates that our droplet contour segmentation algorithm was highly effective, accurately segmenting the contours of the droplets from the original image. Figure 2c shows the droplet uniformity analysis process implemented using the “one-to-three” strategy, yielding a volume coefficient of variation of 2.62%. Figure 2d shows the droplet uniformity analysis process performed using the “one-to-two” strategy, yielding a volume coefficient of variation of 0.94% for the 16 generated droplets. The results revealed that when 16 subdroplets were generated via the “one-to-two” strategy, time savings of 68 s and a 1.67% reduction in the coefficient of variation were achieved relative to the traditional squeezing strategy. Compared with the results of the “one-to-three” strategy, the time savings were 84 s, and the coefficient of variation was reduced by 1.68%. As shown in Fig. 2e, the model could segment a wide range of droplets with varying sizes, colors, morphologies, and brightness levels, which is challenging for conventional algorithms to achieve. Although the model was trained on a transparent droplet dataset, it exhibited good generalizability to colored droplets.

### Success rate calculation

**Fig. 3:**
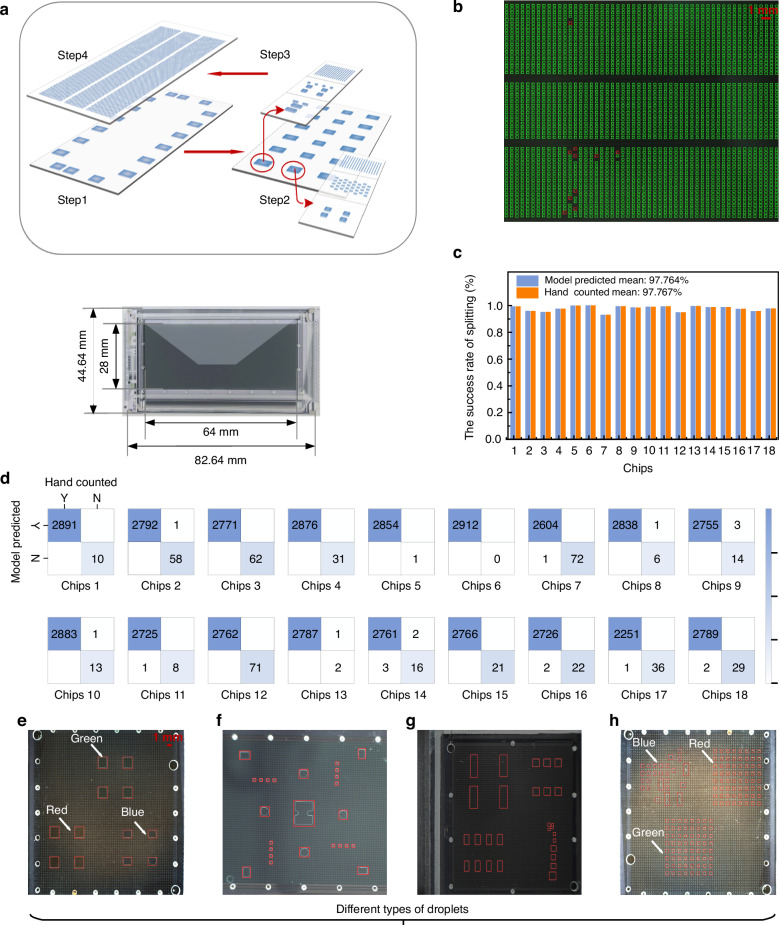
Success rates achieved when intelligently detecting the high-throughput droplet array generation process via the “one-to-two” strategy. **a** Image of the AM-EWOD chip and a schematic diagram of the high-throughput droplet array generation process implemented with the “one-to-two” strategy. **b** The green boxes show droplets that successfully split apart, whereas the red boxes show droplets that did not split apart successfully, as predicted by the model. **c** The success rates of the high-throughput droplet array generation results obtained utilizing 18 parallel AM-EWOD chips were calculated on the basis of both the model predictions and hand counting. **d** Confusion matrices derived from 18 AM-EWOD chips based on the model prediction results. Model performance testing was conducted for different types of droplets. Different colors: (**e**, **h**); different backgrounds: (**f**); different sizes: (**g**); and different brightness levels: (**h**)

Here, we designed a high-throughput AM-EWOD chip containing 640 × 280 electrodes in an active area of 17.92 cm^2^, and these electrodes could be individually or simultaneously addressed (Fig. 3a). We employed the “one-to-two” droplet generation strategy discussed in the previous section to efficiently generate high-throughput droplet arrays. Following a predefined path, the droplets underwent a division process, splitting 5376 subdroplets across the entire pixelated area of the AM-EWOD chip within 369 s (Movie S4), and the process of generating the droplet arrays involved four distinct steps (Fig. 3a). In step 1, we initially injected 18 droplets into the AM-EWOD chip. In step 2, these 18 droplets were subsequently moved to predetermined positions. In step 3, each droplet underwent multiple splits via the “one-to-two” strategy, resulting in multiple subdroplets. Finally, in step 4, 5376 subdroplets with a volume of 2 nanolitres were generated in 369 s.

We used the YOLOv8 model algorithm to automatically calculate the success rate of the high-throughput droplet array generation process. In the “Methods and materials” section, we analyzed the necessity and efficacy of using artificial intelligence algorithms instead of conventional image processing methods for the droplet detection task. The experimental parameter settings are shown in Table S1. During the model training process for droplet detection, the loss functions were calculated in each epoch in the training and validation sets (Fig. S4a–c, d–f). The research community commonly uses the mean average precision (mAP), which combines precision and recall, as the primary metric for conducting performance comparisons among object detection models^48^. The mAP was calculated via the precision‒recall curves produced at various intersection-over-union (IoU) thresholds for the droplets. Precision represents the proportion of true positives relative to the total number of positives, which could be calculated as the number of true positives divided by the sum of the numbers of true positives and false positives. The precision metrics produced by the droplet detection model are shown in Fig. S4g. Recall represents the proportion of true-positive predictions relative to the total number of actual positives, which could be calculated as the number of true positives divided by the sum of the numbers of true positives and false negatives. The recall metrics yielded by the droplet detection model are shown in Fig. S4h. During model training, the mAPs produced at an IoU of 0.5 and within the IoU range of 0.5–0.95 were evaluated for the validation set in every epoch, as detailed in Fig. S4i, j.

ONNX Runtime, an inference framework introduced by Microsoft, was used to deploy the model as software. Through a postprocessing and filtering method based on droplet area thresholds, we intelligently detected the success rate of the high-throughput droplet array generation process (see the software shown in Fig. S5). The model identified the droplets within the red boxes as unsuccessfully split due to their larger areas. Conversely, the droplets within the green boxes were considered successfully generated (Fig. 3b). The software displayed the numbers of red and green boxes in real time, which were used to calculate the success rate of the high-throughput droplet array generation procedure. In practice, we can adjust the area thresholds on the basis of the imposed requirements to filter droplets within different area ranges. For a model to be effectively implemented in the task of intelligently detecting the success rates of high-throughput droplet arrays, its predictions must be stable. Therefore, we manually calculated the success rate of the droplet generation process and compared it with the model-predicted results. Specifically, we selected 18 sets of AM-EWOD chips for testing. For each chip, we manually counted and recorded the numbers of successfully split and unsuccessfully split droplets and then compared these data with the model-predicted results. After completing the manual counting procedure, we input the images of these 18 sets of chips into the model. From the model predictions, we obtained the total numbers of successfully and unsuccessfully split droplets for each set of chips. The mean success rate of droplet splitting was 97.767% as according to hand counting and 97.764% when employing model prediction. The comparative analysis in Fig. 3c shows that the model predictions were highly consistent with the manually counted data, confirming that intelligently detecting the success rate of high-throughput droplet array splitting is highly feasible. The confusion matrix offers an intuitive means of assessing the performance achieved by a model in object detection tasks, especially regarding classification accuracy. It compares the model predictions with the ground-truth labels. The confusion matrices in Fig. 3d show the precision and recall metrics achieved by the model prediction method. Only a few successful labels were incorrectly predicted as unsuccessful, with most predictions being accurate. The model precision was 99.982%, and the model recall was 99.980%. Figure 3e–h display the model predictions obtained for various types of droplets with different colors, backgrounds, sizes, and brightness levels.

### Single-cell recognition

**Fig. 4:**
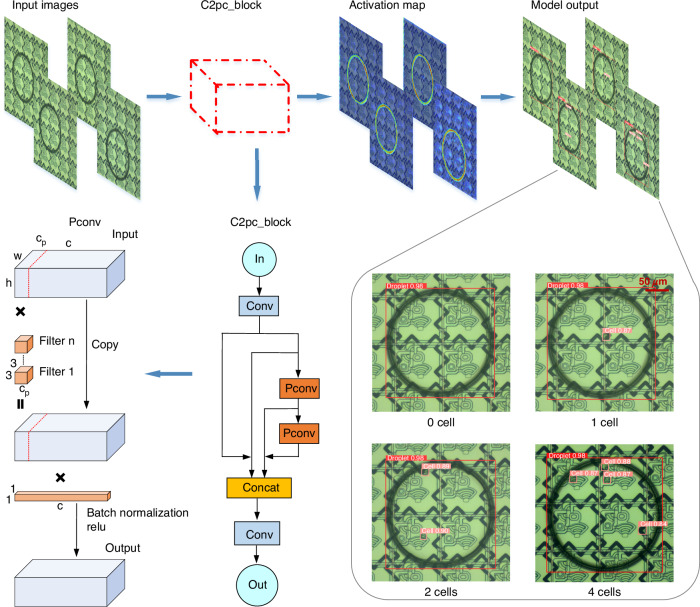
DL workflow for single-cell detection among the droplets. The figure illustrates the procedural steps involved in single-cell detection, including the input image, submodule structure, activation map, and model output. The top-left corner of each box displays the class and confidence score for the corresponding model prediction, whether it is a cell or a droplet

We introduced an improved YOLOv8 detection model for single-cell recognition, addressing the slow speed and low accuracy of the manual single-cell sample detection strategy. Figure S6 illustrates the detailed structure of the cell detection model, and the workflow for the single-cell detection model is shown in Fig. 4. The input dataset was processed by the model, which employed a feature extractor C2pc_block (a cross-stage partial bottleneck with two PCs) and subsequently outputted the results (Movie S5). The C2pc_block, consisting of convolution and partial convolution (PC) components, is a feature extraction module that extracts high-dimensional information from images. The model output consisted of numerous bounding boxes, each containing four spatial coordinates: x and y, representing the center of the box, and w and h, representing the width and height of the box, respectively. Additionally, each box had two class probabilities (p1, p2) and a confidence value indicating the likelihood that the proposed box corresponded to a droplet or cell. Multiple bounding boxes might exist for a single droplet after performing thresholding, so the nonmaximum suppression technique was used to select the bounding box with the highest confidence value. The final bounding box coordinates were saved, and model predictions were then compared with the actual labels, after which the loss function was computed. During model training, the loss functions were calculated in each epoch for the training and validation sets (Fig. S7a–c, d–f). The mAP was calculated via the precision‒recall curves produced under various IoU thresholds for the droplet and cell classes, after which the results were averaged across the droplet and cell classes. During model training, the mAPs achieved at an IoU of 0.5 and IoUs within the range of 0.5–0.95 were evaluated on the validation set in every epoch, as detailed in Fig. S7g, h. Additionally, the precision and recall metrics are provided in Fig. S7i, j.

We evaluated our method on the cell dataset and compared our results with those of four other models: YOLOv5, YOLOv7, Object Box, and YOLOv8. The experimental parameter settings of all five models are shown in Table S1. Table S2 and Table 1 compare the results of this work and those of the representative models. Our method consistently outperformed YOLOv5, YOLOv7, and Object Box, featuring higher mAPs across the training, validation, and test datasets. Table S3 shows the results of a comparison between the proposed method and YOLOv8 combined with different modules on the cell dataset. Compared with the high-performing YOLOv8 model, our method reduced the number of model parameters by 2.5 million and the number of FLOPs by 5.8 billion (Table S2). The model inference time required on the CPU decreased by 15.2 ms, whereas the mAP achieved at an IoU of 0.75 increased by 0.4%, and the cell AP achieved at an IoU of 0.75 increased by 0.7% on the test dataset (Table 1).

**Table 1:** Results of a comparison with other object detectors on the cell dataset

Class	Model	AP^test^_50_	AP^test^_75_	AP^test^
All _(droplets and cells)_	YOLOv5-s	98.2%	95.1%	88.6%
	YOLOv7-tiny	97.9%	93.6%	87.2%
	YOLOv8-s	98.3%	95.4%	89.2%
	Object Box	**98.4%**	94.6%	88.5%
	This work	**98.4%**	**95.8%**	**89.3%**
Improvement		-	+0.4%	+0.1%
Cell	YOLOv5-s	97.0%	90.8%	77.9%
	YOLOv7-tiny	96.3%	87.7%	75.0%
	YOLOv8-s	97.4%	91.5%	**79.4%**
	Object Box	97.4%	89.8%	77.5%
	This work	**97.5%**	**92.2%**	**79.4%**
Improvement		+0.1%	+0.7%	-

The bold values in Table 1 indicate the highest AP scores for each class

Previously, we successfully generated a high-throughput droplet array on an AM-EWOD chip, totaling 5376 droplets. These droplets on the chip were divided into three sections, top, middle, and bottom sections, with 1920, 1536, and 1920 droplets, respectively. Utilizing the improved YOLOv8 model, we individually detected and counted the cells in these 5376 droplets. The output of the model is shown in Fig. 4. Boxes predicted as droplets by the model are marked in red, whereas those predicted as cells are marked in pink. The top-left corner of each box displays the class and confidence score obtained for the corresponding model prediction, whether it is a cell or a droplet. Most droplets contained zero, one, two, or three cells, with only a few containing more than 3 cells. To ensure the accuracy of the algorithm, a manual verification was also conducted. We manually counted and recorded the number of cells in each droplet on the AM-EWOD chip, enabling a comparison with the model prediction results to validate the cell detection capabilities of the developed model.

**Fig. 5:**
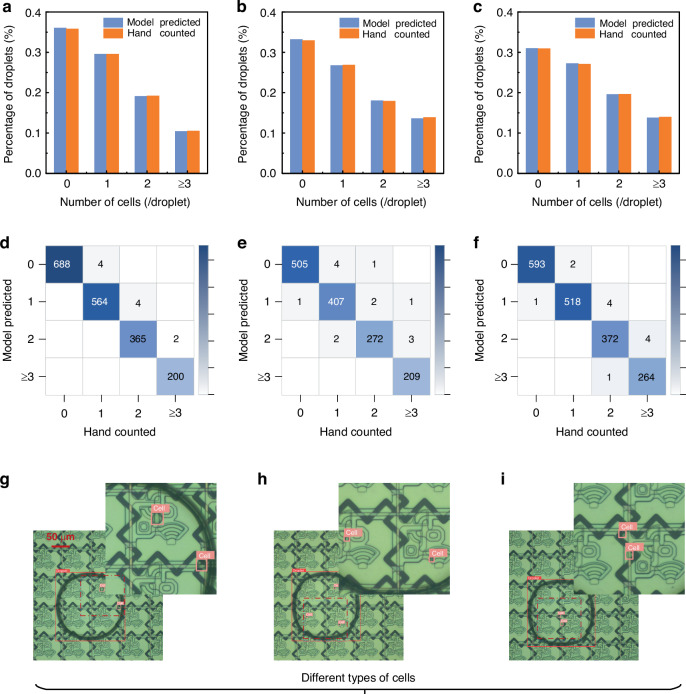
Intelligent detection results obtained for droplets containing a single cell in the droplet array. The percentages of droplets in the top, middle, and bottom sections of the AM-EWOD chip containing zero, one, two, or more than two cells are shown. The droplets were counted on the basis of the model predictions and manual counting. Top: (**a**); middle: (**b**); bottom: (**c**). The confusion matrices derived from the top, middle, and bottom sections of the AM-EWOD chip on the basis of the model predictions are shown. Top: (**d**); middle: (**e**); bottom: (**f**). Model generalizability testing was conducted for different types of cells with different sizes and morphologies. Hybridoma cells: (**g**); mouse spleen cells: (**h**); peripheral blood mononuclear cells: (**i**)

**Fig. 6:**
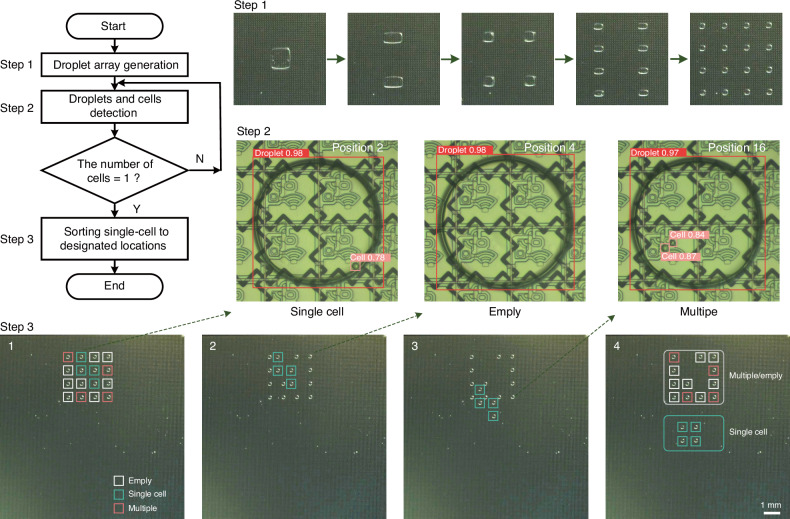
Single-cell droplet sorting and routing experiments. Path-planning results obtained for assigning single-cell droplets to designated locations after they were identified

The results of the model prediction and hand-counting processes are shown in Fig. 5a–c. The x-axis represents the droplets containing zero, one, two, or more than two cells, whereas the y-axis represents the total percentages of these droplets. The cell detection results yielded by the model for the top, middle, and bottom regions of the chip closely aligned with those of manual counting, indicating that our model can replace experimenters in cell recognition tasks. The confusion matrices in Fig. 5d–f show the precision and recall metrics attained for the model prediction results. Most predictions were correct, with only a few labels being incorrectly predicted. In the final analysis, among the 5376 droplets, we successfully identified 1502 single-cell samples, yielding a single-cell generation rate of 27.9%. After conducting a manual verification, the model achieved a precision rate of 99.260% and a recall rate of 99.193% for single-cell detection. To assess the generalizability of the cell detection model, various types of cells with different sizes and morphologies were tested. Figure S8 shows the feasibility of the proposed recognition approach for reflective electrodes and transparent electrodes. For humans, reflective electrodes can provide better optical performance and further assist in the quantification of data for colorimetric analyses. For machine recognition algorithms, it is feasible to identify droplets and cells on different TFT substrates. Figure 5g–i show the model predictions obtained for hybridoma cells, mouse spleen cells, and peripheral blood mononuclear cells. Although the model was trained on a hybridoma cell dataset, it demonstrated good performance on mouse spleen cells and peripheral blood mononuclear cells. As shown in Fig. 6 and Movie S6, we implemented a path-planning system for assigning single-cell droplets to designated locations following their identification. Initially, we split a group of 4 × 4 droplet arrays on the AM-DMF chip. We subsequently employed the improved YOLOv8 model to detect single-cell samples within nanolitre droplets. Finally, we planned a path to assign single-cell droplets to designated locations after they were identified.

## Conclusion

In this work, we addressed the challenges associated with conducting automated biosample determination on an AM-EWOD system for completing various tasks by employing a range of DL models. These models demonstrated optimal performance and generalizability across different types of data, confirming the potential for utilizing AI in other tasks related to AM-EWOD systems. First, we tested the performance of three droplet generation strategies: the traditional squeezing, “one-to-three”, and “one-to-two” strategies. We designed droplet generation paths for these strategies, and the U-Net model was used to automatically evaluate the uniformity of the drops. The results indicated that the “one-to-two” strategy excelled in terms of uniformity, efficiency, and chip utilization during droplet generation. We subsequently used the “one-to-two” strategy to generate a high-throughput droplet array. The YOLOv8 model calculated the success rate of the high-throughput droplet array generation process, achieving an average splitting success rate of 97.76% across 18 tested AM-EWOD chips. A manual verification revealed that the model had a precision rate of 99.982% and a recall rate of 99.980%, effectively overcoming the inefficiency and inconsistency issues associated with manual counting. Additionally, we introduced an improved YOLOv8 model for recognizing single-cell samples in a high-throughput droplet array. A comparison between our results and those of four other models revealed that the improved YOLOv8 model outperformed the competing approaches. We identified 1502 single-cell samples in 5376 droplets, with a single-cell sample generation rate of 27.9%. A manual verification revealed that the model precision was 99.926%, and the model recall was 99.193%, demonstrating its ability to implement high-throughput single-cell sample generation. Finally, we developed a path-planning system that assigns single-cell droplets to designated locations after they are identified. By integrating AM-EWOD technology with DL, we can automatically determine high-throughput biosamples, advancing DMF research into a new era of full automation.

## Methods and materials

### Data collection and composition

In this work, the original datasets were obtained from experimental images and videos. We divided each dataset into three distinct subsets: a training set, a validation set, and a test set. This approach ensured that the model had good generalizability, prevented overfitting, and provided a basis for model selection and parameter optimization. The samples of each dataset were randomly split, with 60% allocated to the training set, 20% assigned to the validation set, and 20% allocated to the test set. The training set primarily served for model weight adjustment and training processes. The validation set was used for model selection and hyperparameter tuning to monitor the performance achieved by the model during training. The test set was designed to evaluate the final performance of the model on unseen data. A summary of the utilized datasets is provided in Table 2, which includes the total numbers of labels and images for the droplet segmentation, droplet detection, and cell detection datasets. The droplet segmentation dataset included 3234 images and 17,178 labels. The droplet detection dataset comprised 5083 images and 6,854,377 labels. The cell detection dataset included 4046 images, 4046 droplet labels, and 5658 cell labels. The ground-truth annotations for the droplet segmentation, droplet detection, and cell detection data used as training examples are shown in Fig. S9.

**Table 2:** Model annotation summary for the utilized datasets

Data/Label	Total count	Training	Validation	Testing
Droplet segmentation dataset				
Droplets	17,178	10,151	3,428	3,599
Images	3234	1940	647	647
Droplet detection dataset				
Droplets	6,854,377	4,111,796	1,380,298	1,362,283
Images	5083	3049	1017	1017
Single-cell detection dataset				
Droplets	4046	2429	810	807
Cells	5658	3402	1100	1156
Images	4046	2428	809	809

Training: validation: testing ratio = 0.6:0.2:0.2

### Conventional and DL method selection and analysis

**Fig. 7:**
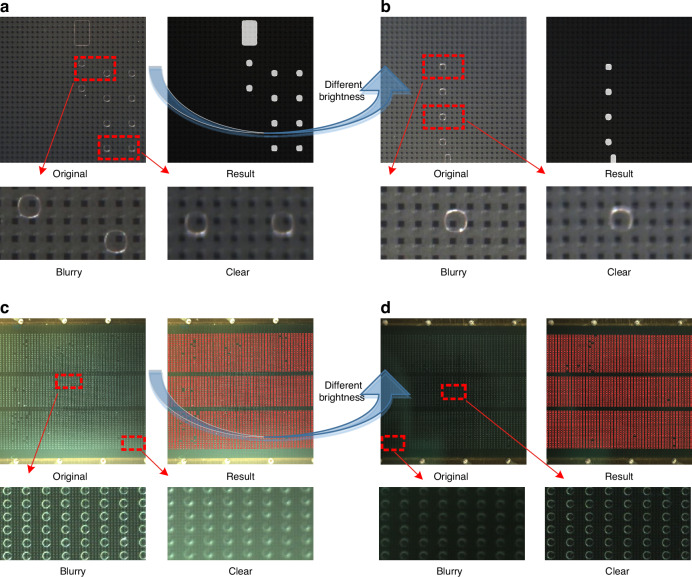
Data analysis results produced for the different tasks involved. The images obtained during the droplet segmentation task (**a**, **b**) and the detection task (**c**, **d**) were captured under various lighting conditions

In practical applications, it is challenging to maintain complete consistency and stability in experimental environments, making it difficult for conventional image processing algorithms to obtain a fully automated solution. Conventional algorithms have limited generalizability, as they rely heavily on manually designed features or predefined distributions. For example, variations in lighting conditions (Fig. 7a, b (original images), Fig. 7c, d (original images)) often require algorithmic parameters, such as threshold ranges, to be adjusted. In addition, droplet outlines vary in terms of clarity at different positions within the same field of view (Fig. 7a–d: clear and blurry images), requiring adjustments to the algorithm parameters, such as the number of erosion and dilation iterations. Consequently, traditional image processing algorithms face challenges when attempting to achieve stable on-chip automation for droplet segmentation and recognition tasks. In contrast, artificial intelligence algorithms demonstrate greater stability and generalizability. As shown in Figs. 2e, 3e–h, and 7a–d, the DL model can segment and detect various types of droplets with different sizes, colors, morphologies, backgrounds, and brightness levels, which is difficult for conventional image processing algorithms to accomplish.

### Model architecture

U-Net is a DL-based segmentation algorithm with a significant influence on the field of image segmentation. The model features a four-layer encoder network and a four-layer decoder network, which converts predictions back to the image pixel domain.

YOLOv7 introduced several advanced techniques, including extended efficient layer aggregation networks, model scaling for concatenation-based models, and planned reparameterized convolution^48^. Object Box was presented as a novel single-stage anchor-free object detection approach^49^. YOLOv8, released in January 2023 by Ultralytics, is a single-stage object detection algorithm. All these model frameworks consist of three primary components: a backbone, a neck, and a head. The backbone, which uses the cross-stage partial (CSP) module, integrates a convolutional neural network to collect and construct image features at various detail levels^50^. The neck uses a path aggregation network (PAN) module and extracts features from the backbone for the head^51^. The head then uses the feature maps acquired from the neck to predict the bounding boxes of objects.

As shown in Fig. S6, we integrated a PC into the C2f module (a cross-stage partial bottleneck with two convolutions) of YOLOv8, creating a new module called a C2pc_block (see the C2pc_block architecture in Fig. 4)^52^. Additionally, we added the coordinate attention (CA) module above the spatial pyramid pooling-fast (SPPF) module in the YOLOv8 model^53^. The C2pc_block helped reduce the complexity of the model and efficiently extract features. While AM-DMF chip electrodes exhibit high background complexity, their positions remain relatively fixed. The CA module enhanced the ability of the model to distinguish between electrode and cell features. An analysis of the effectiveness of the CA module is shown in Table S4.

### AM-EWOD system setup

The utilized AM-EWOD system (DM sys), developed by Guangdong ACXEL Micro & Nano Tech (Foshan, China) and ACX Instruments Ltd (Cambridge, UK), consisted of four main components: an AM-EWOD chip, a core electronic control board, custom-designed control software, and an optical detection system. The optical detection system comprised both high- and low-magnification lenses modeling MV-CS200-10GC and MV-CS050-10GC from Hikvision, primarily for image acquisition purposes. The low-magnification lens was utilized for intelligently analyzing the uniformity of different droplet generation strategies and for intelligently detecting the success rate of the high-throughput droplet array generation process, whereas the high-magnification lens was employed mainly for intelligently detecting cells.

### Reagents and materials

The design of the AM-EWOD chip is illustrated in Fig. 1b. The oil medium used in this study was silicone oil (2cSt) from Dow Corporate. PC 61 5.3 cells were cultured in a cell culture incubator (5% CO_2_, atmosphere, 37 °C). The growth medium for the PC 61 5.3 cells was DMEM containing 10% FBS and 1% Pen Strep. The concentration of the PC 61 5.3 cells was 5 × 10^5^ cells/ml. The PC 61 5.3 cells [PC 61; PC 61.5.3] (CL-0663) were obtained from Procell Life Science & Technology Co., Ltd.

## Supplementary information

supplementary information clean

Movie S1

Movie S2

Movie S3

Movie S4

Movie S5

Movie S6

## Data Availability

The authors declare that all relevant data are available in the paper and its Supplementary Information Files, or from Z.J. (2021200184@mails.cust.edu.cn) on request.

## Code availability

The source code, pre-trained weights, and samples associated with this paper are available in GitHub via the repository (https://github.com/li-an666/DL_AM-DMF). Support and more information are available from Z.J. 2021200184@mails.cust.edu.cn.
