# Supplementary material for: Artificial intelligence-enabled multipurpose smart detection in active-matrix electrowetting-on-dielectric digital microfluidics
Source: Microsyst Nanoeng. 2024 Sep 27;10:139. doi: 10.1038/s41378-024-00765-7 (PMC11427566; doi:10.1038/s41378-024-00765-7)
Supplement: Supplementary file 1 — supplementary information clean [file 41378_2024_765_MOESM1_ESM.pdf]

Supplementary Materials for

Artificial intelligence-enabled multipurpose smart detection in active-matrix digital electrowetting-on-dielectric digital microfluidics

Zhiqiang Jia[1,2,3], Chunyu Chang[2,3], Siyi Hu[2,3], Jiahao Li[4], Mingfeng Ge[2], Wenfei Dong[1,2*], and Hanbin Ma[2,3,4*]

Correspondence to: wenfeidong@sibet.ac.cn and mahb@sibet.ac.cn

This PDF file includes:

Figs. S1 to S9
Tables S1 to S4
Movies S1 to S6

Fig. S1.

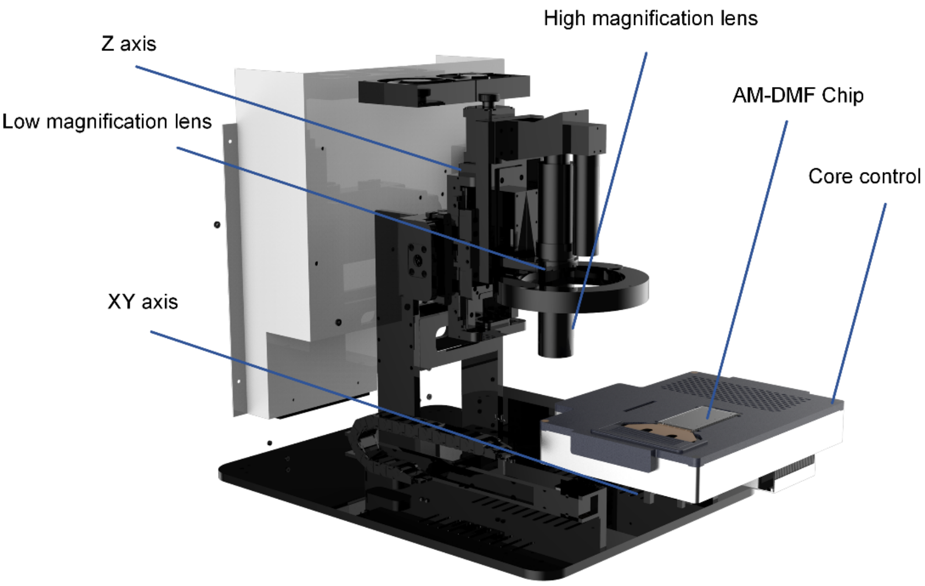

Fig.S1. The structure of the DM sys.

Fig. S2.

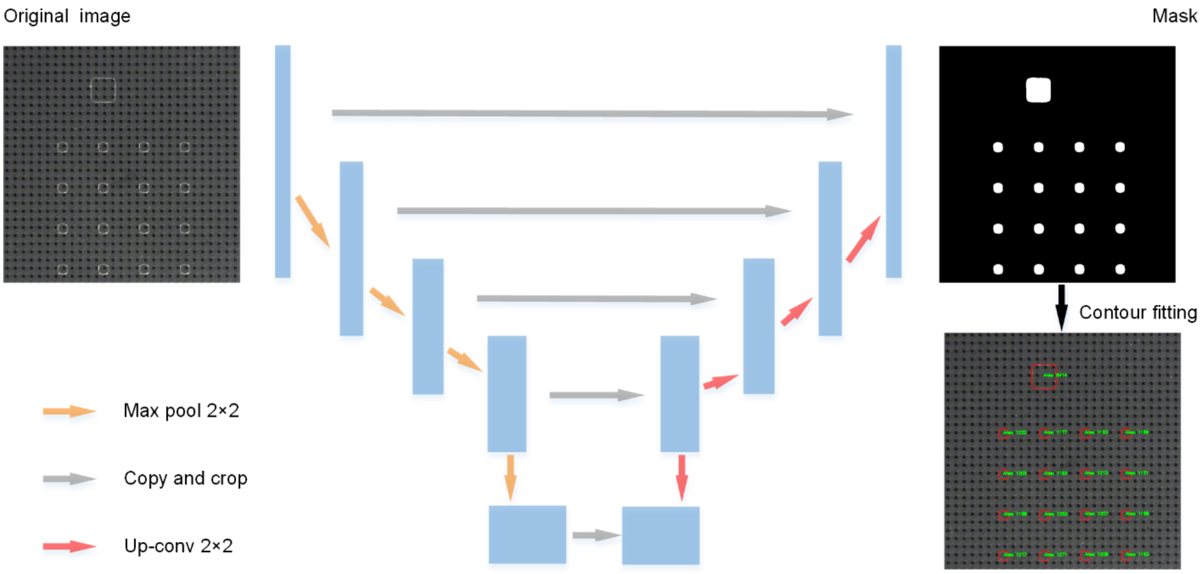

Fig.S2. Deep learning workflow for droplet segmentation model.

Fig. S3.

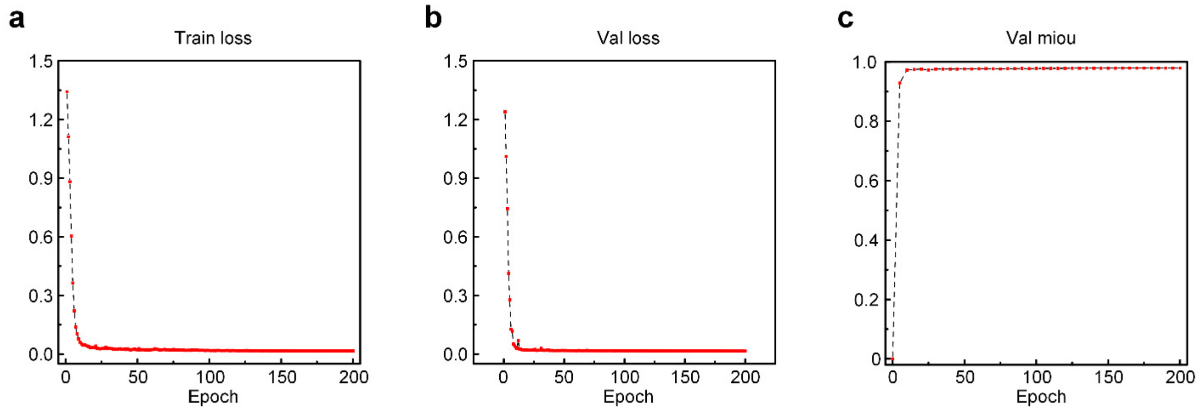

Fig.S3. The performance of the droplet segmentation model on the training and validation set. The loss function of the model on the training set (**a**) and validation set (**b**). (**c**) The mIoU function on the validation set.

Fig. S4.

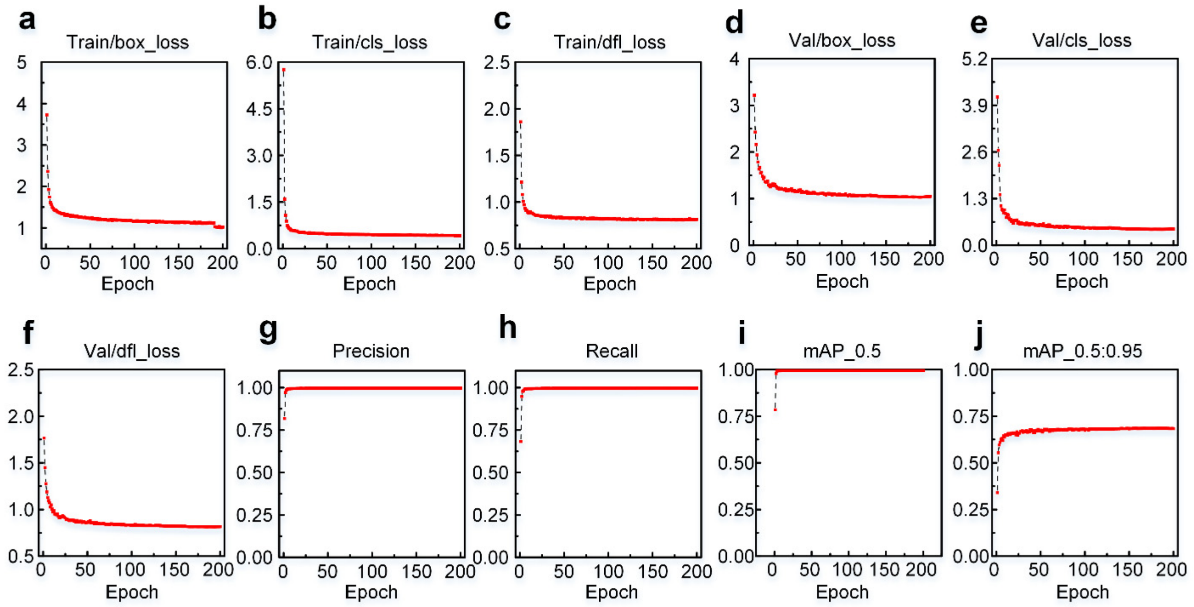

Fig.S4. The performance of the droplet detection model on the training and validation set. The loss function of the model on the training set (**a-c**) and validation set (**d-f**). The precision metric (**g**), the recall metric (**h**), the map at 0.5 iou (**i**), and the range of 0.5–0.95 iou (**j**) of the model on the validation set.

Fig. S5.

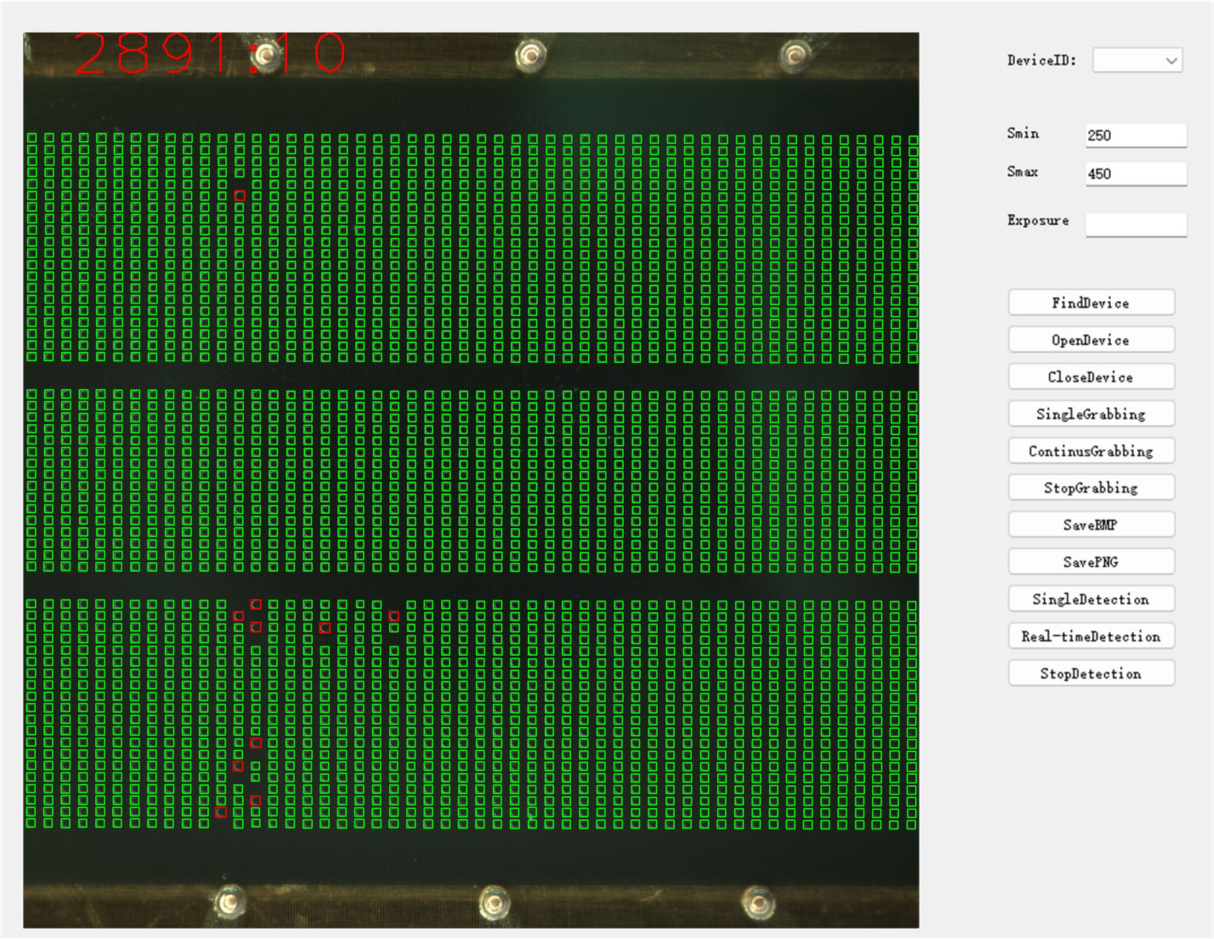

Fig.S5. Software for intelligent detection of success rate in high-throughput droplet array generation.

Fig. S6.

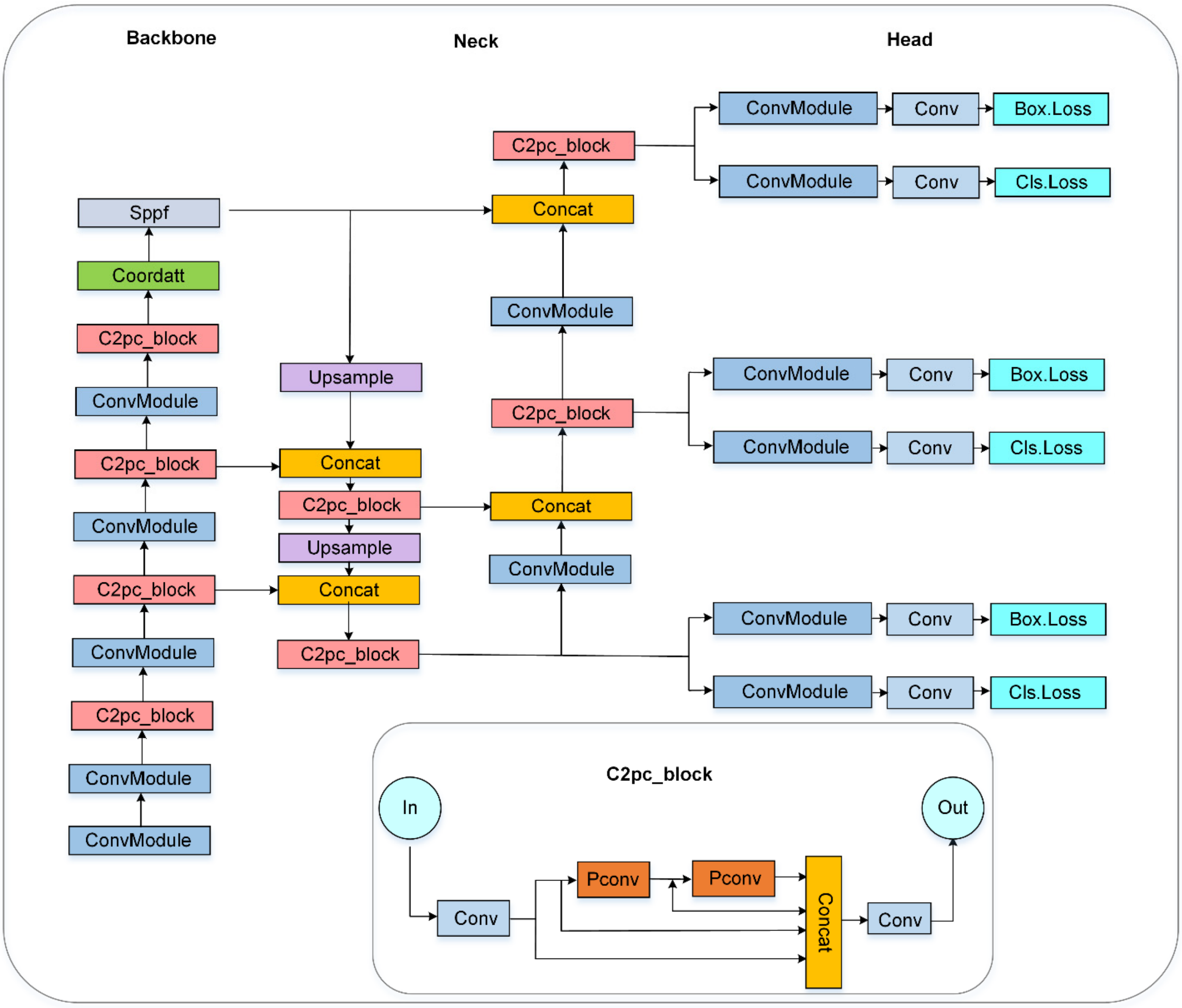

Fig.S6. The structure of the cell detection model.

Fig. S7.

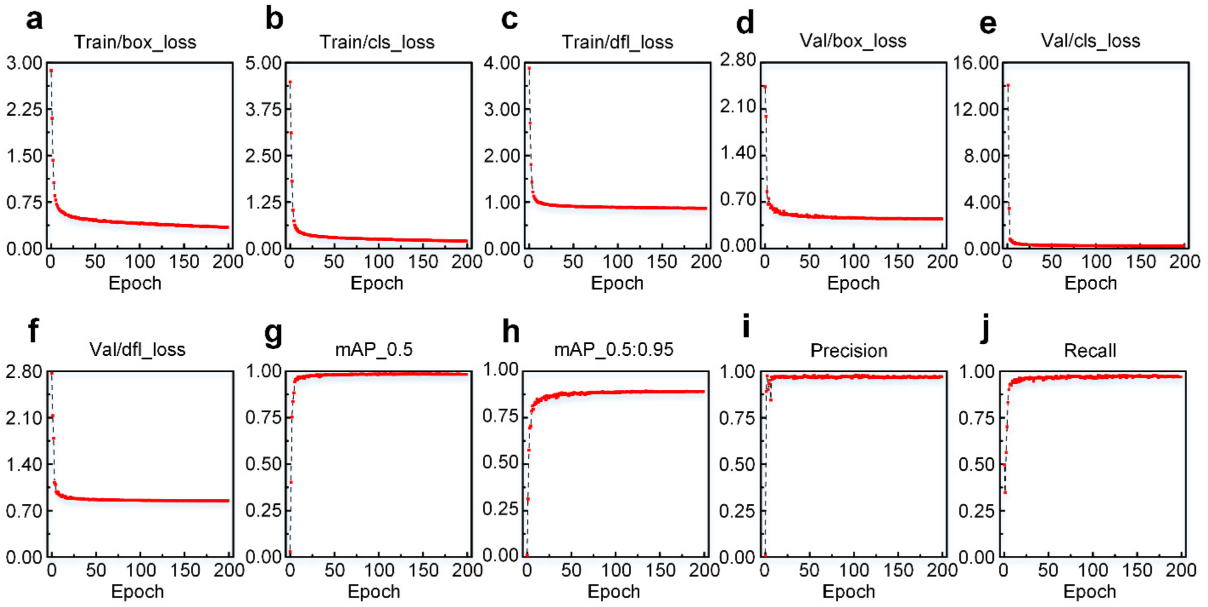

Fig.S7. The performance of the cell detection model on the training and validation set. The loss function of the model on the training set (**a-c**) and validation set (**d-f**). The map at 0.5 iou (**g**), and the range of 0.5–0.95 iou (**h**), the precision metric (**i**), and the recall metric (**j**), of the model on the validation set.

Fig. S8.

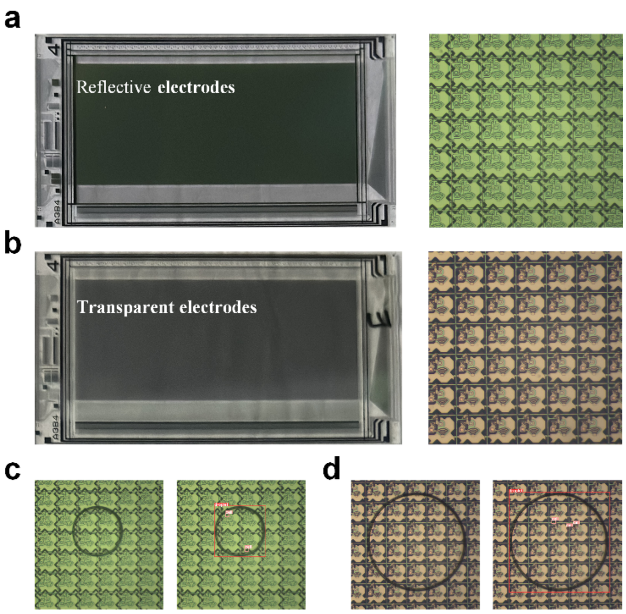

Fig.S8. Model performance testing on reflective electrode plates and transparent common electrodes. (**a**), Reflective electrodes. (**b**), Transparent electrodes (**c, d**) Model predicted on different TFT substrates.

Fig. S9.

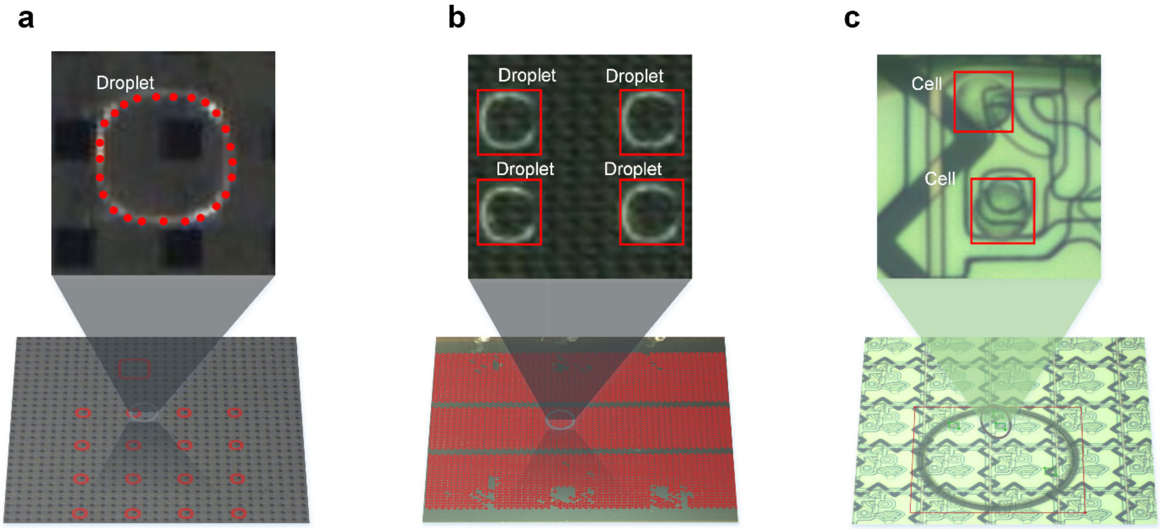

Fig.S9. Data composition. Ground truth annotation for the droplet segmentation model (**a**), droplet detection model (**b**), and cell detection model (**c**) were used as training examples.

Table S1. Model parameter settings for droplet segmentation model, droplet detection model, and cell detection model.

Droplet segmentation model	Droplet detection model	Cell detection model
Device: 3090-24 GB	Device: 3090-24 GB	Device: 3090-24 GB
Image size: 896	Image size: 1024	Image size: 1024
Batch size: 4	Batch size: 16	Batch size: 32
Initial learning rate: 0.0001	Initial learning rate: 0.005	Initial learning rate: 0.01
Momentum: 0.9	Momentum: 0.937	Momentum: 0.937
Weight decay: 0	Weight decay: 0.0005	Weight decay: 0.0005
Freeze epoch: 50	Warm-up epochs: 3	Warm-up epochs: 3
Maximum epochs: 200	Warm-up momentum: 0.8	Warm-up momentum: 0.8
Optimization: ADAM	Maximum epochs: 200	Maximum epochs: 200
Number of classes: 1	Patience: 50	Patience: 50
	NMS threshold: 0.6	NMS threshold: 0.6
	Optimization: SGD	Optimization: SGD
	Number of classes:1	Number of classes:2

Table S2: Comparison with other object detectors on the dataset.

Models	Parameters	FLOPs	Size	Speed (ms)
YOLOv5-s	7.0 M	16.0 B	1,024	273.7
YOLOv7-tiny	6.0 M	13.2 B	1,024	252.4
YOLOv8-s	11.1 M	28.6 B	1,024	358.4
Object Box	7.0 M	15.9 B	1,024	274.6
This work	8.6 M	22.8 B	1,024	343.2

Table S3: Comparison with different modules on cell dataset.

Models	AP_{50}^{test}	AP_{75}^{test}	AP^{test}	Parameters	Speed
YOLOv8	97.4%	91.5%	**79.4%**	11.1 M	358.4 ms
YOLOv8+C3Ghost[1]	96.7%	89.8%	77.5%	**5.9 M**	**257.6 ms**
YOLOv8+RepC3[2]	97.1%	91.7%	79.3%	15.7 M	463.3 ms
YOLOv8+BottleneckCSP[3]	97.1%	91.2%	79.3%	10.9 M	353.1 ms
YOLOv8+CBAM[4]	97.4%	91.2%	79.2%	11.2 M	362.8ms
YOLOv8+DCNv2[5]	93.9%	81.9%	67.9%	11.9 M	370.7 ms
YOLOv8+SimAM[6]	97%	91.4%	79.3%	11.1 M	361.7 ms
This work	**97.5%**	**92.2%**	**79.4%**	8.6 M	343.2 ms
Improvement	+0.1%	+0.7%	-	-2.5 M	-15.2 ms

Table S4: Analysis of the effectiveness of the CA module.

Models	AP_{50}^{test}	AP_{75}^{test}	AP^{test}
YOLOv8+C2pc_block	97.0%	91.1%	79.2%
YOLOv8+C2pc_block+CA	97.5%	92.2%	79.4%
Improvement	+0.5%	+1.1%	+0.2%

Movie S1.
Traditional squeezing droplet generation strategy**.**
Movie S2.
"one-to-three" droplet generation strategy.
Movie S3.
"one-to-two" droplet generation strategy.
Movie S4.
High-throughput droplet array generation.
Movie S5.
Droplets and cells detection.
Movie S6.
Sorting single-cell droplets to designated locations.

References

1. Tang, Y. et al. GhostNetv2: Enhance cheap operation with long-range attention. *Advances in Neural Information Processing Systems* **35**, 9969-9982 (2022).
2. Soudy, M., Afify, Y. & Badr, N. RepConv: A novel architecture for image scene classification on Intel scenes dataset. *International Journal of Intelligent Computing and Information Sciences* **22**, 63-73 (2022).
3. Wang, C.-Y. et al. CSPNet: A new backbone that can enhance learning capability of CNN. *Proceedings of the IEEE/CVF conference on computer vision and pattern recognition workshops*, 390-391 (2020).
4. Woo, S., Park, J., Lee, J.-Y. & Kweon, I.S. Cbam: Convolutional block attention module. *Proceedings of the European conference on computer vision (ECCV)*, 3-19 (2018).
5. Zhu, X., Hu, H., Lin, S. & Dai, J. Deformable convnets v2: More deformable, better results. *Proceedings of the IEEE/CVF conference on computer vision and pattern recognition*, 9308-9316 (2019).
6. Yang, L., Zhang, R.-Y., Li, L. & Xie, X. Simam: A simple, parameter-free attention module for convolutional neural networks. *International conference on machine learning*, 11863-11874 (2021).
